# Advanced Multi-Dimensional Cellular Models as Emerging Reality to Reproduce *In Vitro* the Human Body Complexity

**DOI:** 10.3390/ijms22031195

**Published:** 2021-01-26

**Authors:** Giada Bassi, Maria Aurora Grimaudo, Silvia Panseri, Monica Montesi

**Affiliations:** Institute of Science and Technology for Ceramics, National Research Council of Italy (ISTEC-CNR), 48018 Faenza, Italy; giada.bassi@istec.cnr.it (G.B.); aurora.grimaudo@istec.cnr.it (M.A.G.)

**Keywords:** multicellular spheroids, organoids, organ-on-a-chip, nanostructured biomaterials, tissue engineering, 3D *in vitro* models

## Abstract

A hot topic in biomedical science is the implementation of more predictive *in vitro* models of human tissues to significantly improve the knowledge of physiological or pathological process, drugs discovery and screening. Bidimensional (2D) culture systems still represent good high-throughput options for basic research. Unfortunately, these systems are not able to recapitulate the *in vivo* three-dimensional (3D) environment of native tissues, resulting in a poor *in vitro–in vivo* translation. In addition, intra-species differences limited the use of animal data for predicting human responses, increasing *in vivo* preclinical failures and ethical concerns. Dealing with these challenges, *in vitro* 3D technological approaches were recently bioengineered as promising platforms able to closely capture the complexity of *in vivo* normal/pathological tissues. Potentially, such systems could resemble tissue-specific extracellular matrix (ECM), cell–cell and cell–ECM interactions and specific cell biological responses to mechanical and physical/chemical properties of the matrix. In this context, this review presents the state of the art of the most advanced progresses of the last years. A special attention to the emerging technologies for the development of human 3D disease-relevant and physiological models, varying from cell self-assembly (i.e., multicellular spheroids and organoids) to the use of biomaterials and microfluidic devices has been given.

## 1. Introduction

The *in vitro* reproduction of the human body is an exciting and arduous challenge for medical doctors, biologists and bioengineers who have tried to resemble the complex mechanisms undergoing in healthy and pathological tissues for decades. Therefore, the fundamental question is: “How do we capture the human biological complexity in robust translational *in vitro* models?” [1]. In addition, the pharmaceutical industry is looking for new opportunities to significantly accelerate and improve drug discovery. The drug discovery and development process (DDDP) is affected by a high financial impact of drug candidates’ failures. Indeed, the United States (USA) or European Union (EU) pharmaceutical companies invested in 2016 approximately 18.3% and 14.4% of their own annual net turnover, respectively, in the research and development (R&D) phase of DDDP, with an increasing trend in the last years. In 2017, the member states of the EU spent together almost € 320 billion on R&D with a high failure rate [2,3]. This limitation has driven the industry demand and the clinical need for the development and validation of more predictive *in vitro* disease-relevant models to be used during the preclinical phase discovery.

Conventional 2D cell cultures still represent the gold standard models for *in vitro* research [4] as simple, cost effectiveness, robust and a good high-throughput practice option for cell-based biology and pathogenesis studies [5]. Nevertheless, these models have well-recognized inadequacy in recapitulating the 3D environment of human tissues composed of different cell types, tissue-specific ECM, biological factors and interactions with neighboring cells [6], resulting in a poor *in vitro-in vivo* correlation.

The introduction of 2D co-culture systems partly resembles the cell population heterogeneity of *in vivo* tissue thanks to the possibility of investigating cell–cell interactions and intracellular communication cues in a fast, simple and economically feasible way [7]. However, the absence of a relevant and tissue specific-ECM is still missing, making the 2D co-culture unreliable. It is well-known that several factors affect the cellular behavior in both physiological and pathological process, such as the ECM architecture, composition, mechanical properties, kinetics of biomolecules release, vascular perfusion and the several interactions among these elements. For example, in 2D standard models cells are always cultured on a higher stiffness surface (i.e., ~105 kPa plastic Petri dish) compared to lower ECM stiffness of soft tissues (i.e., 1–25 kPa) and, consequently, different cytoskeletal components adjustments are induced [8], directly affecting cellular polarity, metabolism and protein expression [6].

In addition, the *in vivo* preclinical–clinical translation process is affected by several limitations of animal models that often could not consider factors such as gender and age in the prediction of the human responses to the new therapies [9], without forgetting the increasing ethical concerns of inevitable sacrifice of animals [10].

**Figure 1:**
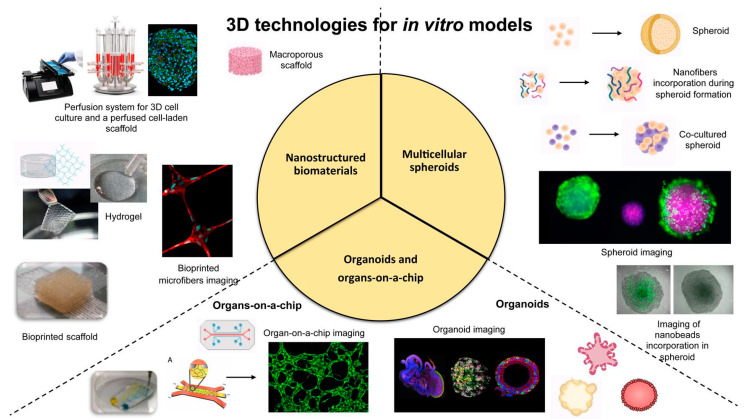
Schematic representation of the most promising technologies and tools for the engineering of 3D *in vitro* models.

Dealing with these challenges, scientific research has recently focused the attention on the design and bioengineering of innovative *in vitro* 3D approaches that (i) provide optimal and promising platforms to closely capture *in vivo* microenvironment in a laboratory setting, (ii) seal the gap between the 2D culture systems and animal models, (iii) improve tests outcomes and, eventually, (iv) decrease animals use for *in vivo* studies. Several versatile tools and technologies are currently employed to develop 3D *in vitro* reliable models, such as cell self-assembly (i.e., multicellular spheroids and organoids), nanostructured scaffolds and hydrogels and microfluidic devices (i.e., organ-on-a-chip and bioreactors) as summarized in Figure 1; most important, the interdisciplinary and dependency on each other of available approaches from various fabrication techniques are indispensable for the creation of the most mimetic model of the required tissue (Figure 1).

The evolutionary step from 2D to 3D models has introduced tremendous biomimetic features [11], such as the ability in recapitulating tissue-specific ECM in terms of physico-chemical properties and more specific cell responses, resembling the highest complexity of the human body. The increasing demand for these 3D culture systems is also reflected in a strong economic growth of a new market segment dedicated to the *in vitro* cell evaluation products [12].

The present review has been focused on the most advanced progresses in the field of 3D approaches for the development of innovative models of physiological and pathological tissues and organs highlighting advantages and limitations. The emerging technologies for the development of human 3D disease-relevant and physiological models, from cell self-assembly to the use of biomaterials and microfluidic devices, will be discussed in an attempt to give to the lecturer an exhaustive overview of recent trends regarding the innovative technology used in this research field.

## 2. Multicellular Spheroids

Multicellular spheroids defined as cellular aggregates growing as spheres derived from the intrinsic self-assembly of cells suspended in biological fluids or in a matrix better reflect the networks affecting functionality, viability, polarity and protein secretion of cells, leading to a more realist microenvironment compared to 2D culture conditions [13,14].

Spheroids obtained from different techniques are already suitable for basic physiology and metabolism studies as well as for tumor biology, toxicology studies and the development of bio-artificial tissues due to the high reproducibility and low related-costs [15]. A variety of approaches from scaffold-free non-adherent surfaces or hanging drops to the recent introduction of microfluidic devices, microfabricated platforms and Magnetic Levitation Method (MLM) are available for spheroids production [12,16]. However, technical hurdles include variable size, poor control on cell viability, functions and differentiation within the spheroid, the absence of extracellular matrix, and the presence of a decreasing gradient of nutrients and oxygen from the outside to the core of the spheroid. Cells composing the spheroid, with particular attention for those at the outer surface, are generally compactly organized and their functions result inhibited by limited diffusion of biomolecules and other factors crucial for cell proliferation and differentiation [17]. Moreover, a stable spheroid size is relevant and desirable during drug testing, as it can directly affect the bioactivity of the studied substances [18].

Facing these drawbacks, spheroids were recently manufactured with biomaterial-based techniques including micro/nanobeads (e.g., gelatin microparticles [19], magnetoferritin nanoparticles [20] and gold and iron oxide nanoparticles [21,22]) and nanofibers (i.e., (poly (L-lactide) (PLLA) fibers [23] and poly(lactic-co-glycolic acid) (PLGA) fibers [24]), hydrogels (e.g., spheroid embedding in collagen/fibrin hydrogel [25], alginate hydrogel [26] and polyethylene glycol (PEG) hydrogel microspheres [27]) and functionalized cell membranes (e.g., fibronectin/gelatin nanofilm [28], boronic acid copolymer application [29]). 

### 2.1. Micro-Molded Non-Adherent Surfaces and Hydrogels 

Among scaffold-free techniques, the use of non-adherent surfaces is a cost-effective and easy method for spheroid production consisting on the seeding of a cell suspension onto a non-adhesive substrate (e.g., agarose [30] and poly(2-hydroxethyl methacrylate) (HEMA) [31,32]) that promotes cellular aggregation preventing cellular adhesion to substrate [33,34]. For example, Ahmad T. et al. [23] describes the incorporation of homogenously distributed mineralized fragmented nanofibers during adipose-derived stem cells (hADSCs) spheroidization process on 1.3 wt % agarose gel-coated plate to obtain a 3D *in vitro* osteochondral tissue from spheroids fusion. The use of non-adherent gel surface together with fibers, prepared by fragmentation of PLLA mineralized with different concentrations of sodium bicarbonate (0.005, 0.01 and 0.04 mM) simulated body fluid, allowed to fabricate relatively loosely assembled spheroids with enhanced intra-spheroid diffusion of nutrients and osteogenic cues; indeed, large spaces created among cells by fibers incorporation leads to spheroid with reduced cell arrangement and increased cell functions, viability and growth of cells into the vacant spaces of the spheroid. The presence of sodium bicarbonate played a critical role in crystal growth and mineral grain size, where 0.04 mM concentration led to the most homogenously and apatite-mimetic tightly coating nano-minerals [17,23]. However, the degradation rate of nanofibers into spheroids was not reported by the authors; their possible degradation over time could induce again close cellular adhesion blocking nutrients diffusion within the spheroid undoing the beneficial effects, or it could positively affect cell behavior due to the release of bioactive degradation products.

Besides the advantages of using non-adherent surfaces, limitation in size and shape of resulted spheroids is problematic [35]. In this context, micro-molded non-adherent surfaces can be obtained by design software and microfabrication techniques to form individual spheroids with different and controllable shapes, size and cell functions [36,37]. As an example, in 2020, Yarali et al. [38] reported the spheroidization process of human umbilical vein endothelial cells (HUVECs) with soluble integrin-binding (Arg, Gly and Asp) peptide to form an *in vitro* scaffold-free microtissue (SFM) of *in vivo* pre-vascularized structure. The use of non-adhesive molds supported the safe and controllable formation of endothelial spheroids by preventing cell adhesion to the gel and promoting cells spontaneously self-assembling. Moreover, the spheroidization of endothelial cells with 2 mM RDG peptide by simple dilution in EGM-2 bullet media showed the highest level of living cells, vasculogenic maturation related-genes and protein expression (Vascular Endothelial Growth Factor (VEGF), Tyrosine-protein kinase receptors such as Tie-1, Tie-2, Vascular Endothelial Cadherin (Ve-Cadherin) and Platelet Endothelial Cell Adhesion Molecule (PECAM-1)) compared to other concentrations (0, 1 and 4 mM), confirming the role of the peptide in promoting vasculogenesis by integrin-mediate cell interactions and vascular markers induction [39,40]; indeed, the native reorganizing ability and functions of HUVECs with up to 2 mM RDG concentration were comparable to cells embedded in bulk materials, such as hydrogels [38,41], demonstrating the obtainment of a pre-vascularized microtissue without any material support [42]. 

Alternately, hydrogels as spheroids-embedding molds can help to control their size, rate of growth, drug responses and creating a more realistic model of *in vivo* situation [43]. For example, Utama et al. [44] established a custom high-throughput bioprinting method for producing alginate and calcium chloride hydrogel-embedded spheroids of three different tumor cell lines (neuroblastoma, non-small cell lung cancer and glioblastoma cells) with controlled spatial distribution and size obtaining statistically reliable data in comparison to others research works. Briefly, the authors proposed a bespoke drop-on-demand 3D bioprinter able to print a high density of cells in a single droplet directly into a hydrogel mold using a solenoid microvalve printhead. An alginate cup was firstly printed in a 96-well plate and then a cell-laden ink with 250 million cell/mL printed into the cup; inside the matrix, a combination of gravitational forces and ECM secretion by cells caused the formation of single embedded spheroid for each well in 24 h. Interestingly, as the cup was filled by the growing spheroid, the latter conformed to the shape of the cup confirming the bioprinter ability to controlled shape by matching cup size, cell volume and density in each droplet together with relevant tumor-like properties [44]. 

### 2.2. Magnetic Levitation 

The application of external forces (e.g., electric [45] or magnetic field [46], ultrasounds [47]) facilitates cell aggregation by the integration of stimuli-responsive biomaterials, liposomes or nanoparticles [48]. Among these, MLM involves the cell incorporation of magnetic particles subsequently embedded in hydrogels; during cell culture, the exposure to negative magnetophoresis allows magnetic cells to live suspended against gravity and aggregate [21,49,50]. For example, Lewis et al. [22] developed a spheroid model composed by Mesenchymal Stem Cells (MSCs) and magnetic iron oxide nanoparticles implanted into a type I collagen gel resembling the stiffness of *in vivo* human bone marrow niche (modulus of 36 Pa of the proposed model [22,51] versus the modulus of 100 Pa of in vivo niche [52]). The exposure to a magnet induced the self-assembly of magnetic MSCs into spheroids with a specific phenotype, providing a potential platform of *in vitro* stem niche of bone marrow. 

With the same principle, Labusca et al. [53] exploited magnetic levitation to obtain hADSCs spheroids with enhanced stemness properties and subsequently possible application for regenerative medicine. Indeed, magnetic nanoparticles are deeply investigated for biomedical applications as they can be easily controlled by magnetic field responsiveness. Successfully, hADSC loaded with nanoparticles (derived by a mechano-chemical hydrothermal approach based on ferrous sulfate heptahydrate and ferric chloride hexahydrate) formed aggregates with increased volume, cell viability, proliferation and mobility. Most important, spheroids stemness was confirmed by cell ability in three differentiation lineages (ostegenic, adipogenic and chondrogenic), with remarkable adipogenic conversion. 

Besides the promising results, some authors have demonstrated changes in cellular structures and possible apoptosis by external forces and artificial gravity manipulation [48]. 

### 2.3. Microfluidic Devices 

Microfluidics flows cells through sub-millimeter microchannels into compartmentalized micro-chambers separated by semipermeable barriers where micro-rotational flows induce cell aggregation [54]. This technique is suitable for high throughput production of spheroids thanks to the presence of biosensors that monitor flow rates in real-time [55,56]. Undoubtedly, this approach can serve as bioreactors for biomimetic stimuli, for assuring both mechanical (e.g., fluid flow) and chemical cues (e.g., oxygen gradients), to the micro-engineered tissues [57]. 

A mimetic engineering approach was performed by Park et al. [58] by the incorporation of microfluidic device to exploit fluid flow emulating the interstitial flow of cerebrospinal fluid and investigate neurological diseases such as Alzheimer’s disease. Indeed, the interstitial fluid is fundamental for delivering nutrients and eliminate metabolic wastes thought and from the brain tissue [59]. On this line, the authors used a microfluidic chip with 50 cylindrical concave microwells for the formation of prenatal rat cortical neurospheroids treated with 5 µM amyloid-β (Aβ) for three days, while an interstitial flow was applied simultaneously by an osmotic pump able to maintain the slow speed range of interstitial flow typical of the brain tissue (from about 0.1 to 0.3 µL/min) [60]. The reinforced neural network by fluidic flow and the infiltration of Aβ into the neurospheroid definitely suggested that the integration of spheroids and microfluidic technology could yield an *in vitro* model with more relevant physiological outcomes and controlled experimental conditions [61]. Recently, the same authors improved the previous model including microglial cells capable of inducing a neuroinflammatory microenvironment [62], obtaining a more complex and more predictive *in vitro* system called 3D organotypic human Alzheimer’s disease culture model.

Most importantly, microfluidics facilitates drug penetration into spheroid models [63,64], as successfully reported by Shi et al. [65]. The authors describe the successful transport of different concentrations of paclitaxel (1, 3, 10, 30, 100, 300, and 1 μM) though blood vessels within a 3D *in vitro* tumor model by the application of a bilayer microfluidic device. An apical and a basal polydimethylsiloxane (PDMS) layers within overlap channels and separated by a semipermeable membrane for constant nutrients supply composed the microfluidic device. The presence of blood vessels was mimicked by a monolayer of endothelial cells (EC) on the membrane and a cell-laden Matrigel of human colon cancer cells (HTCC116) was seeded on the basal channel to form the complete 3D vascular-tumor model. In this way, tumor spheroids successfully formed due to constant flow of nutrients and media from the apical channel up to the basal one though the membrane, while metabolic wastes were removed from the apical channel outlet by the reserve path. Paclitaxel testing on the model showed a remarkable efficacy of the drug on tumor spheroids; most important, the endothelial layer not only mimicked the blood vessels with their typical permeability and drug resistance but also act as indicator of the toxicity of the drug on healthy cells [65,66].

The potential is well-known of the pluripotency of human induced pluripotent stem cells (hiPSCs) in regenerative medicine [67]. In this context, the spheroidization process can be exploited for inducing the differentiation of hiPSCs in various cellular lineages [68] as reported by Hirano et al. [69]; the authors obtained hiPSCs aggregates in a non-adhesive agarose-gel microwell plate by a fluidic channel-based culture system able to maintain serially differentiation-specific culture media. The combination of simple microwells with fluidic system allowed the obtainment of aggregates with controlled size by simply regulation of cellular density in the injected suspension (224 ± 13 mm with 1250 cells/well, 275 ± 14 mm with 2500 cells/well and 338 ± 16 mm with 5000 cells/well). Additionally, pancreatic endocrine cells were obtained from hiPSCs; endoderm lineage (DE), pancreatic endoderm (PE) and immature pancreatic islet cells (II) were subsequentially induced during 3, 7 and 10 days of culture and verified by successful analysis of gene expression profiles and flow cytometry DE (SRY-box transcription factor 17- SOX17, forkhead box A2- FOXA2), PE (SRY-box transcription factor 9- SOX9, pancreatic and duodenal homeobox 1- PDX1), II (NK6 homeobox 1- NKX6.1). Undoubtedly, the authors provided an *in vitro* low-cost and suitable system for scalable and rapid obtainment of functional somatic cells from hiPSCs typically required in regenerative medicine and transplantations. 

### 2.4. Multi-Approached Methods 

The hanging drop method creates spheroids by the placement and subsequent aggregation of cells at the bottom of a droplet suspension due to gravity and meniscus at the air-liquid interface. The method is frequently used due to its simplicity in obtaining homo- and heterotypic spheroids [70]. On the other hand, spheroids are confined in single drops, without communication, exchange of media and metabolites with other ones [71]. Several authors proposed combinations of the method with other emerging approaches to improve its outcomes. 

In 2014, Frey et al. [71] presented a versatile *in vitro* analytical platform derived by advancements of the conventional hanging drop method with microfluidic device incorporation to create fluidic and interconnected networks of hanging drops. This concept provided (i) controllable spheroids of different cell types on the same platform, (ii) a fluidic interconnection among spheroids, (iii) the constant control on nutrient and drug dosage and (iv) the incessant spheroids communication to create complex multi-organ models. The microfluidic device was composed by rim-micropatterns on the surface of a PDMS substrate that defined limited areas and rim-distance for size drop control. As a novel concept, the fluidic conduits were designed and run on the completely open system to guarantee gas exchange and specific fluid dynamics compared to typically closed devices. Clearly, this concept offers completely new perspectives about multicellular spheroid culture [71].

The spheroid model also gained much popularity in studying *in vitro* subpopulations of stem-like cells present in certain tumor masses, named cancer stem cells (CSCs), that show stemness properties as self-renewal [72,73,74], resistance to conventional therapies [75] and clonal growth in free-floating spheres, called tumorspheres [76,77]. In this context, the most popular spheroids growing techniques (i.e., free-floating spheres and multi-cellular tumor spheroid model (MCTS)) are used to isolate and unravel CSCs presence in a wide variety of tumors, including brain, breast, lung and head and neck squamous cell carcinoma (HNSCC). However, free-floating spheres required prolonged cultivation time and many manipulation steps. Additionally, MCTS guaranteed the partial enrichment of CSCs due to presence of fetal bovine serum (FBS) supplementation in the culturing medium that has a well-known differentiation effect, making them not usable for high-throughput demands [78]. In this context, Gorican et al. [78] proposed an innovative *in vitro* spheroid model for CSC-enrichment, named SCESM (Stem Cell Enriched Spheroid Model), suitable for high-throughput screenings of CSC-specific compounds for HNSCC by combining the more specificity of free-floating spheres and the working convenience of MCTS methods. Similar medium conditions of free-floating cultures (i.e., Endothelial Growth Factor (EGF) and basic Fibroblast Growth Factor (bFGF) concentrations) were used to support CSCs proliferation, over-expression of stemness genes and proteins (i.e., CD44, CD73, CD90 and CD133) and a faster diameter increase (i.e., from 450 to 800 µm in just one day of culture) for guaranteeing the formation of a hypoxic area and necrotic core within the spheroid [79]. Similar to the MCTS method, the authors seeded a high concentration of cells (3500 cells/well) in a round-bottom ultra-low attachment (ULA) 96-well plate for a rapid formation of large, compact, uniform and spatially separated spheroids with a proper nutrient gradient, obtaining spheroids uniformity, stability in size and growth pattern similar to solid tumors [80]. Despite the cheap and effective CSC enrichment method suitable for high-throughput screening (HTS), the complexity of the surrounding microenvironment should be considered for the investigation of the detected substances in a more real culture [78,81].

## 3. Organoids and Organs-on-a-Chip

### 3.1. Organoids

The presence of basilar anatomic microstructures and functions render organotypic platforms interesting for their application as 3D *in vitro* models. Among these organotypic structures, organoids can be defined as miniaturized organs with a 3D structure and multiple cell layers of tissue-specific cell types and stem cells within a unique intrinsic organization [82]. In this context, several organoid types have been investigated for mimicking skin, intestine, liver, kidney, lung, pancreas, and brain, and some examples will be discussed in detail. The possibility to use organoids for modeling pathology and diseases renders these systems promising for studying some aspects of pathogenesis and new targets discovery [55]. For example, organoid cultures have been employed for modeling infectious diseases of the gut [83], stomach [84], and in cystic fibrosis [85]. However, different disadvantages related to the use of organoids have to be mentioned: (i) the small size that renders these systems difficult to manage; (ii) the inadequate nutrients supply; (iii) the insufficient exchange of gases and (iv) and unproper removal of cellular waste products. Importantly, the actual manufacturing technology for organoids shows poor reproducibility, rendering the application of such systems in pharmaceutical studies challenging [86]. Moreover, these systems show biochemical gradients of soluble factors produced by cells which are not controlled and do not resemble the graded distribution observed *in vivo*. Concerning the organoids growth, the sole presence of passive diffusion for exchanging nutrients, oxygen and waste substances renders these systems unable to support their growth and maturation [87].

The research is very active in ophthalmic, where organoids have been already used to investigate new drug candidates *in vitro* for the treatment of macular degeneration, glaucoma, cataracts and several retinal disorders, such as age-related macular degeneration (AMD) or diabetic retinopathy. Among these models, retinal organoids for retinal disease investigation have been developed by self-assembling of layers of differentiated photoreceptors [88,89]. Additionally, reported retinal organoids were demonstrated to be responded to light in a similar way to neonatal retina [90].

Common neural disorders include traumatic brain injury (TBI), spinal cord injury (SCI), Parkinson’s disease, Alzheimer’s disease, Huntington’s disease and neurodevelopmental disorders as autism. Understanding the mechanisms of such Central Nervous System (CNS) diseases requires platforms capable of properly mimicking *in vitro* the *in vivo* neuronal environment. In this regard, organoids can be used to determine how different neuronal subtypes interact with each other to cause the different pathologies [91]. From this perspective, brain organoids resembling discrete areas can be considered as a promising platform for investigating neural development, as well as neurodevelopmental or neurodegenerative diseases [92]. Various human brain organoids have been proposed using self-organizing 3D stem cell cultures [93,94,95]. As an example, Lancaster and colleagues [93] successfully reproduced the human forebrain using PLGA copolymer fiber microfilaments as a floating scaffold, demonstrating neuroectoderm formation, cortical development and characteristic cortical tissue architecture. Similarly, Paşca et al. [94] differentiated pluripotent cells *in vitro* to study normal and abnormal corticogenesis by simply generating a laminated cerebral cortex–like structure. Successfully, Renner and colleagues [95] recapitulated *in vitro* forebrain organizing centers, demonstrating the timed generation of neurons with mature morphologies, astrocytes and oligodendrocytes.

### 3.2. Organs-on-a-Chip

As reported, the lack of vascularization, a homogeneous distribution of multiple cell types and the absence of tissue specific cell densities of common 3D models represent some serious challenges of organoids. Partly overcoming these limitations, organs-on-a-chip have been designed as micro-structured platforms to mimic functional units of human organs *in vitro* [96]. Interestingly, organs-on-a-chip provide different advantages over organoids, such as the presence of cell–cell interactions, spatio-temporal gradients of chemicals and mechanical strain and vasculature-on-a-chip mimicking the interstitial flow, although the overall costs and reproducibility are still challenging [97]. Interestingly, organ-on-a-chip are also able to resemble the biochemical and biomechanical cues of ECM [86]. Lastly, the use of organs-on-a-chip allows a higher density of systems culturing, rendering these systems more reproducible and easier to manage [87]. 

Organotypic structures can be generated organizing cells in a spatially-precise manner by 3D-bioprinting and assuring a controlled and disease-specific tissue irroration by microfluidic devices [98]. Interestingly, 3D bioprinting was shown to generate proper cellular models because cells can be loaded at high densities. For these reasons, 3D printing is used by surgeons to replicate the detailed anatomy of organs prior to surgery [99,100,101,102,103,104,105], and for the construction of relevant *in vitro* models for pharmaceutical applications [106]. Relevant studies regarding organ-on-a-chip systems reported in the review are summarized in Table 1. 

**Figure 2:**
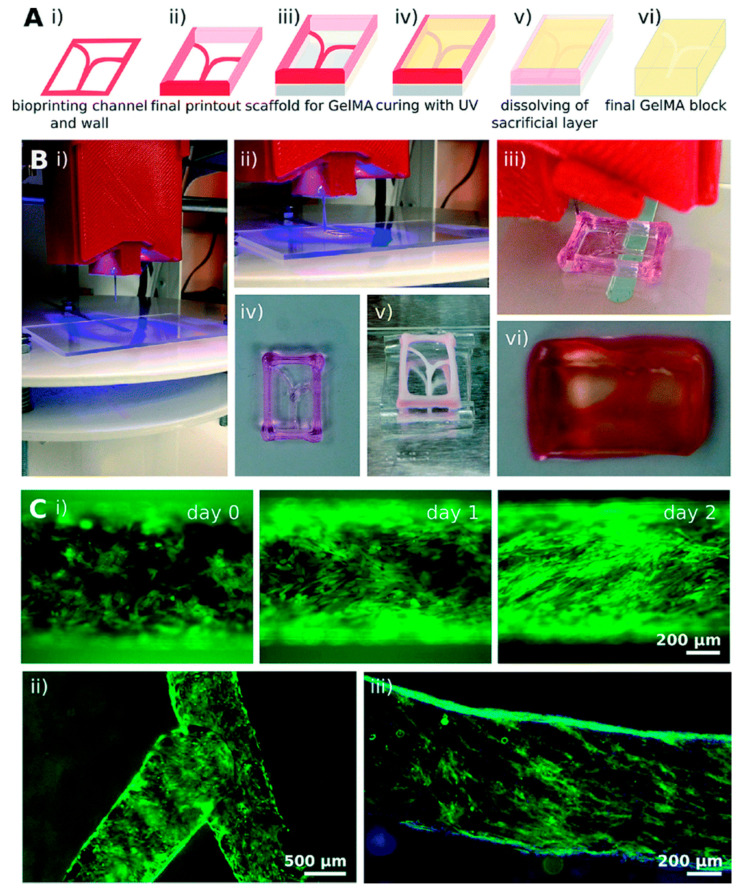
Thrombosis 3D bioprinted model designed by Zhang and coworkers from [108] with permission. Sacrificial bioprinting of vascularized hydrogels. (**A**) Schematic representation of the bioprinting process steps (i–vi) and (**B**) corresponding photographs of: (i) and (ii) bioprinting of a Pluronic template; (iii) template drying placed on a PDMS support; (iv) GelMA filling and ultraviolet crosslinking; (v) dissolution of the sacrificial channels; (vi) final construct with hollow channels. (**C**) Visualization of hollow microchannels endothelialization inside the GelMA construct: (i) linear and (ii) bifurcating microchannels; (iii) CD31 (green) and nuclei (blue) staining of the confluent layer of HUVECs.

Organs-on-a-chip have been also designed associating microfluidics to 3D printing techniques. The advantage connected to such an association is the possibility of creating a biomimetic heterogeneous microenvironment associating direct cell printing with complex 3D microstructures. In this regard, fluid flow in organ-on-a-chip models is expected to reflect physiological flows such as blood flow, and tissue functions like peristalsis, breathing and heartbeat [107]. Concerning this aspect, microfluidic devices have been already demonstrated effective for serving as vascular models and vascularized systems. As an example, Zhang and colleagues [108] successfully used 3D bioprinting technology for the construction of a highly biomimetic thrombosis-on-a-chip model consisting of microchannels coated with a layer of confluent human endothelium embedded in a gelatin methacryloyl (GelMA) hydrogel (Figure 2). Successfully, authors demonstrated that the encapsulation of fibroblasts in the designed GelMA matrix caused deposition of collagen type I over time, facilitating fibrosis remodeling and suggesting that the 3D bioprinted model could be successfully employed for studying thrombosis.

As an example, Wang and colleagues 3D bioprinted a functional cardiac tissue mimicking the native myocardium using a fibrin-based composite hydrogel as bioink [109]. Successfully, spontaneous and synchronous contraction was observed in the bioprinted model, while positivity to Actn1 (Alpha-actinin-1) and Cx43 (connexin 43) proteins indicated the presence of aligned and coupled cardiac cells. In another study, Zhang et al. [110] designed an anisotropic endothelium layer by mixing a composite alginate bioink, endothelial cells and rat-derived cardiomyocytes. Interestingly, an aligned, spontaneously and synchronously contracting tissue has been generated and then this tissue has been embedded in a microfluidic perfusion bioreactor to create a myocardium-on-a-chip.

Park et al. [111] developed the first bioprinted lung-on-a-chip using ECM bioink derived from tracheal mucosa, demonstrating a higher expression of vascular markers, as well as adequately induced inflammatory responses useful for studying dust-mite-induced exacerbation *in vitro*. Another interesting example is the eye-on-a-chip developed by Seo et al. that provided a realistic platform to study dry eye disease [112]. In details, these authors used primary human keratocytes and epithelial cells, a 3D cell culture scaffold coupled with a perfusion chamber, a tear channel and a biomimetic eyelid. Lastly, dry eye disease has been induced by the authors by fixing the frequency of blinking actuation at six times per minute to simulate reduced blinking rates observed *in vivo* in patients.

Interestingly, complex organs-on-a-chip models of intestine have been engineered including neighboring channels lined by human microvascular endothelium, immune cells, and pathogenic bacteria. As an example, Shah and coworkers [113] designed a modular microfluidics-based human–microbial co-culture model, HuMiX, to mimic gut microbioma, demonstrating the possibility of building a host-microbiome ecosystem containing Caco-2 cells and anaerobic human gut bacteria by a constant perfusion of culture medium.

**Figure 3:**
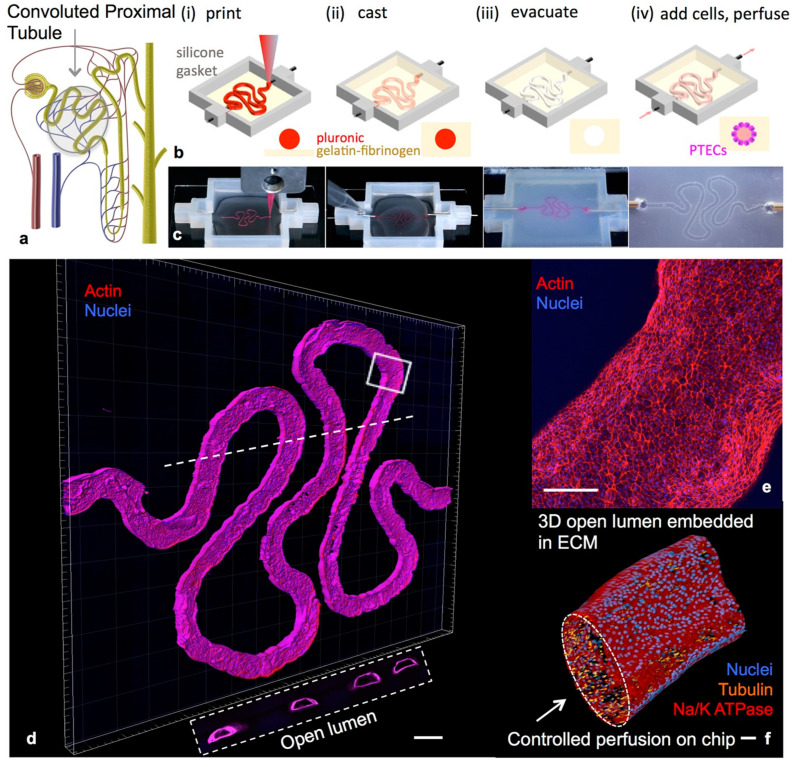
Example of a 3D convoluted renal proximal tubule on chip [114]. Schemes of a nephron highlighting the convoluted proximal tubule (**a**) and different steps in the fabrication of the 3D model (**b**,**c**); a 3D rendering of the printed convoluted proximal tubule acquired by confocal microscopy (**d**–**f**). PECTs: proximal endothelial tubule cells

Homan et al. [114] associated bioprinting to microfluidic technologies for developing a renal-proximal-tubule-on-a-chip model (Figure 3). This model structure has been created by printing Pluronic^®^ F127 onto a gelatin–fibrinogen matrix at first step, which was then liquefied at 4 °C for allowing cell seeding and perfusion. Interestingly, the proximal tubule epithelial cells seeded in the 3D model showed a well-defined polarization and produced kidney-specific cytokines, resembling *in vivo* situation.

Still now, the investigation of the mechanisms of the myelotoxic stress induced by radiation or drugs is challenging because of the inaccessibility of this tissue *in vivo*. For this purpose, Chou et al. [115] designed a vascularized bone marrow-on-a-chip system composed by a channel filled with a fibrin gel, CD34+ cells and bone marrow-derived stromal cells, and a parallel channel covered by human vascular endothelium and perfused with culture medium. Thanks to this system, the authors successfully recapitulated the myeloerythroid toxicity typically observed after exposures to chemotherapeutics or ionizing radiation.

Organotypic platforms have also been used in tumor modeling. In a recent study, the development of a 3D co-cultured contraction and invasion tumor model has been reported by performing an innovative “organotypic assay”, called “Mini-Organo”. In details, L. Chitty et al. [116] seeded a Mini-Organo solution composed by collagen type I, FBS, CalciNeurin B (CNB) and CAFs (cancer associated fibroblasts) suspension in bovine serum albumin (BSA) coated well plates before polymerization. Then, the same model was used to measure cancer cell local invasion into the CAFs-remodeled matrix by seeding cancer cells on the top of the Mini-Organo and transferring it on stainless steel grid for chemoattract factor. In this way, the authors proposed an innovative tool for higher *in vitro* throughput screenings of molecules and interventions, not only limited to the cancer field, thanks to the versatility of the model using different cell types under different experimental conditions.

Recently, Marturano-Kruik et al. [117] developed a human bone-on-a-chip to investigate metastatic colonization of the bone by different mechanisms of vascularization and drug resistance. The model consisted of human endothelial cells and bone marrow-derived MSCs (hBM-MSCs) cultured in a native bone matrix and placed into a microfluidic chip for cell exposure to fluid flow. Then, the authors mimicked metastatic colonization using a functional human tri-culture system and infusing breast cancer cells. In this situation, slow flows supported the niche by driving oxygen, nutrients and signaling factors from the blood to the interstitial tissue, while ECM, endothelial cells and MSCs regulated metastatic homing. Successfully, breast cancer cells exposed to interstitial flow exhibited a slow-proliferative rate typically associated to drug resistance. Resembling the tissue irroration, the authors proved that a long-lasting, self-assembled stable vascular network supported by MSCs has been obtained without supplementation of any angiogenic factor in a microfluidic-based cancer model with precise biophysical manipulation [117]. In another study, Ma and coworkers [118] developed a leukemia-on-a-chip system to simulate B cell acute lymphoblastic leukemia/bone marrow interactions and for the investigation of the role of bone marrow niche in the regulation of B cell acute lymphoblastic leukemia chemoresistance. In this case, authors successfully demonstrated the use of their model to test niche co-targeting regimens.

In another example, glioblastoma, the most common brain cancer accounting for around 50% of all malignant primary brain tumors [119], has been considered. Yi and collaborators created a 3D glioblastoma-on-a-chip (GBM) model by association of bioprinting, micro-vessels integration and microfluidic oxygen-gradient-generating system [120]. From removing natural cellular components, decellularized pig brain ECM (BdECM) bioink with patient-derived cancer cells and HUVECs has been printed to create a compartmentalized ring-structural scaffold differently seeded in the core and on the outside, respectively. The GBM model was developed into a chamber wall printed with gas permeable silicon ink on a non-permeable glass substrate to create an oxygen radial gradient. Successfully, the authors developed a heterogenous ecosystem of cancer analogue on a chip that simultaneously presented: a biochemical cue (the brain-ECM-like environment) and two biophysical cues (the compartmentalized stroma composed by vascularized section and a radial oxygen-gradient-generating system) [120,121]. The high level of heterogeneity of the model contributed to the emergence of various pathological features of GBM, such as the differential clinical responses to chemoradiotherapy [120]. The efficacy of cancer immunotherapy *in vitro* for the treatment of glioblastoma was investigated by Cui and coworkers [122]. These authors developed a microfluidics-based and patient-specific glioblastoma-on-a-chip system to optimize anti-PD-1 immunotherapy for different tumor subtypes. Successfully, the authors were able to ablate CD163+ M2-tumor associated macrophages, strengthened CD154+CD8+ T-cell functionality and GBM apoptosis by co-administration of CSF-1R inhibitor BLZ945 and nivolumab, rendering this system suitable for a personalized screening of immunotherapies.

## 4. Nanostructured Biomaterials

In recent decades, naturally derived matrices, bio-scaffolds, hydrogels and nanostructured systems have been considered as the most suitable platforms for *in vitro* resembling of ECM with appropriate properties to the native one [121,123,124]. Decellularization of tissues [125], microfluidics [124], electrospinning [126,127], freeze-casting [128] and bioprinting [129] still represent the most frequently used manufacturing techniques for the fabrication of *in vitro* supports. However, a clarification of the advantages, limitations and usefulness of available methods is necessary due to the disparate applications of the different approaches.

### 4.1. Decellularization Process

**Figure 4:**
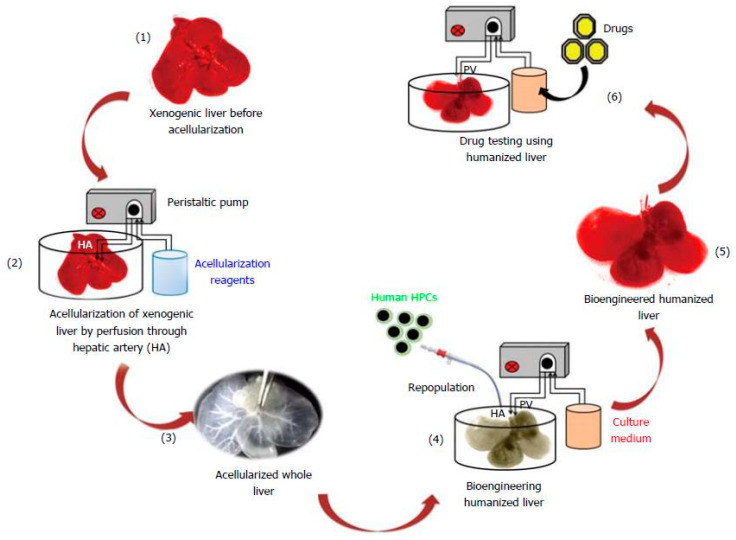
3D perfused humanized liver from [131]. Schematic experimental plan to generate 3D humanized liver by using acellularization and human patient-derived cells repopulation strategy.

The decellularization process aims to remove cellular and nuclear components from the natural tissue by treatment with different agents (chemical, biological or physical) to obtain natural derived matrices with preserved native ultrastructure, ECM components (i.e., various types of collagen, fibronectin, elastin, microfibrils, proteoglycans, glycosaminoglycans (GAGs)) and various growth factors [130]. For example, a perfused humanized liver generated by recellularization of a decellularized liver scaffold with human patient-derived hepatic stem cells was reported by Vishwakarma, S.K. et al. [131] (Figure 4). After recellularization by infusion of media and cells via the main hepatic artery of liver, a natural organization of cells, a high level of liver albumin secretion and urea synthesis were successfully observed together with an intact ECM with well-distributed proteins (collagen, fibronectin, laminin), vasculature and mechanical properties in the immediately post-decellularized liver. Additionally, the analysis of the metabolization of six well-established substrates of enzymes cytochrome P450 (CYP) confirmed the efficient metabolic activity of the recellularized model [131,132]. Similar approaches are available for pancreas [133] and cartilage [134]. Other relevant models based on decellularized matrices concern bone [135], spinal cord [136], kidney [137], cancers [138,139] and also commercially available CorTM PATCH [140] and equine Matrix PatchTM [140] currently used for *in vitro* studies, surgery and repair of cardiac tissue. 

Another application concerns the use of decellularized matrices (dECM) as bioink for printing highly mimetic hydrogels compared to those obtained with available inks for 3D bioprinting [141,142]. Along these lines, dECM-based hydrogels of different organs such as lung [143], kidney [144], brain [145], spinal cord [146], bone [147], colon [148] and umbilical cord [149] have been successfully printed, demonstrating an excellent prediction of therapeutic response, *in vivo*-like framework, extensive application space, abolition of intra-spaces differences and, as consequence, offering a promising chance to replace animal models from human preclinical screenings [125,150]. However, the use of dECMs for biomedical application is still in its initial stage; the scaling up of the decellularization process represents one of the biggest challenges due to the numerous manual operations, the large deviation between different decellularization techniques, the request of standardization and automatization of methods for different tissues and the lack of universal guidelines that make the approach unsuitable and expensive [125,150,151]. Moreover, while physical decellularization, including freezing, pressure, sonication and agitation, causes considerable destruction of the 3D structure, enzymatic approaches could remove matrix components (i.e., fibronectin, laminin and elastic) which are important for cellular differentiation and meanwhile increase the costs owing to the large volumes of proteases, nucleases and chelators required to obtain a significant result [152].

### 4.2. Electrospinning Method 

**Figure 5:**
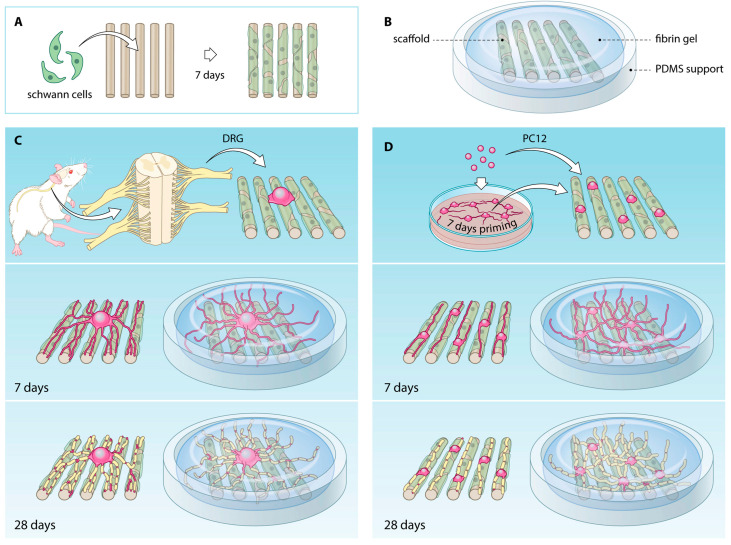
3D PN model from [155]. Formation of bands of Büngner (BoB) by SCs (**A**); overview of the model components (**B**); representation of neural cell population (PC12 or DRG)-SCs co-culture procedure (**C**,**D**).

Electrospinning technology processes solutions or melts of polymers into homogenous fibers with diameter distribution from nanometers to sub-micrometers [127] using a high electric field. Interestingly, nanofibrous scaffolds show highly porous structures with large surface-to-volume ratios that support cell interactions, rendering these systems attractive choice for tissue engineering applications [153]. Microfibrous scaffolds are frequently used in CNS models as they are able to guide axonal growth longitudinally [154]. In this context, Malheiro et al. [155] reported a 3D model of peripheral nerve (PN) of CNS consisted of Schwann cells (SCs) and a neuronal cell population seeding on an aligned electrospun microporous scaffold [154] subsequently embedded in a 3D fibrin hydrogel (Figure 5). The co-polymer PEOT/PBT (Poly(ethyleneoxideterephthalate)/poly(butyleneterephthalate)), already used for nerve conduit fabrication [156], was electrospun to obtain ultrathin scaffolds (12 mm diameter) composed by aligned fibers (1.37 ± 0.20 µm diameter), that were seeded with SCs and neural cells showing anisotropic bands enwrapped around scaffolds and an increased neurite growth, respectively. Moreover, the embedding hydrogel made neurites extend along and above the scaffolds in multiple layers, compared to scaffolds alone [154,155]. Another example reported the electrospinning of a polyaniline-gelatin-polycaprolactone (PANi-GEL-PCL) nanoscaffold superficially modified by plasma method for bone tissue engineering [157], but also for bone regeneration [158], cardiac repair and diabetes [159,160] and aneurysm modeling [161].

It is possible to assert that the electrospinning technique offers the possibility of large-scale production, easy functionalization and material combinations, but at the same time it has been demonstrated that the chaotic fiber deposition could negatively affect cellular response; indeed, fibers with submicron diameters could cause a tightly packed network in which pore sizes could be too small for cells infiltration [162,163].

### 4.3. Freeze-Casting Method

Freeze-casting, also known as ice templating, is a versatile technique to tailor pore structure and mechanical properties by manipulation of parameters (i.e., the degree of porosity, pore size, pore shape and orientation) [128,164,165] during scaffold fabrication according to tissue requirements. In this context, Nematollahi Z. et al. [165] reported a chitosan silk-based scaffold with enhanced mechanical strength for tissue engineering of long segments of the trachea by the adjustment of freezing rate and the amount of the cross-linking glutaraldehyde (GA) agent. Other attempts to reconstruct *in vitro* the trachea were relatively unsuccessful considering the higher mechanical performance of freeze-casted scaffolds in resembling the complexity of human trachea [166]. Thus, the authors combined silk fibroin and chitosan by freeze-casting with three different consolidates rates (0.5, 1 and 2 °C/min) along with three concentration of GA (0, 0.4 and 0.8 wt %), where 1 and 2 °C/min freezing rates and 0.8 wt % GA resulted in a homogenous porous structure having compatible tensile strength and elastic modulus with human trachea [167]. In another case, chitosan-alginate porous scaffold was demonstrated to support cell growth of osteoblasts [168], chondrocytes [169] and embryonic stem cells [170] as the use of solutions with lower acetic acid concentration during freeze-casting promoted a more uniform pore structure and lower solution viscosity [171]. However, the whole biocompatibility and biodegradability of freeze-casted scaffolds are the most restrictive factors [165].

### 4.4. 3D Bioprinting Technology

Recently 3D bioprinting has emerged as promising technique because it provides the control of structure in all X, Y and Z directions during fabrication process thanks to the digital design of the frame using a computer-aided design software or scanning from medical images before printing [172]. Moreover, this technology can directly pattern cells within the material without cells aggregation caused by potentially uncontrolled cell distribution of traditional cell seeding on pre-fabricated scaffold [129]. Bioprinting has recently been successfully applied to neural tissue engineering because it can easily control the mechanical, structural and cellular properties of nervous tissue. Gu et al. encapsulated human neural stem cells (hNCSs) within a hydrogel ink composed of alginate, agarose and carboxymethyl-chitosan (CMC) to form a 3D neural mini-tissue [173]. In details, cell-loaded bioinks of 5% *w*/*v* alginate and different concentrations of agarose (0.5, 1.5 and 2.5% *w*/*v*) and CMC (2, 3.5 and 5% *w*/*v*) were tested to direct-write extrusion printing, and 5% *w*/*v* CMC and 1.5% *w*/*v* agarose resulted the most printable and defined gel construct with uniform cell distribution compared to other concentrations. The successful co-bioprinting of cells and ink offered a simple approach for cell-biomaterial interfacing, where the use of hydrogel platform helped in situ differentiation of hNSCs along with glial cells and neural network formation. On this line, 3D bioprinting can be suitable for incorporating different cell types, bioactive factors or/and macromolecules within bioink to more resemble the complexity and functionality of neural tissue as well as others [173,174,175]. As an example, the spinal cord contains multiple neuronal cell types within an arrangement of gray matter (neurons and motor neurons in dorsal/lateral and ventral roots, respectively) surrounded by ascending and descending white matter with axon tracts carrying afferent and efferent signals [176,177]. In this context, Joung et al. developed a spinal cord platform by using an extrusion-based multi-material bioprinting process to print Matrigel bioinks with specific cell types (spinal neuronal progenitor cells—NPCs and/or oligodendrocyte progenitor cells—OPCs) in precise positions within alginate/methylcellulose printed scaffold [178]. The scaffold ink and the cell-laden bioinks were sequentially printed in a layer-by-layer manner to create multiple channels of ~150 × 300 × 5000 (w × h × l) µm dimensions with precise cell population [179,180].

The authors of the previous existing study suggested the future improvement of the reported model by the incorporation of 3D printed stimuli-responsive filled capsules for creating gradients by programable release within the architecture, promoting neuronal functions and axon guidance [181,182,183]. Interestingly, this concept is possible by the introduction of the “fourth dimension” which involves the application of stimuli-responsive smart biomaterials during common 3D bioprinting process to easily control changes in the architecture or cues gradients within the scaffold. On this line, Betsch et al. [184] reported a successful magnetic-based fiber alignment mechanism to print three different layers of articular cartilage (superficial layer in close contact to synovial fluid, the middle and the deep one in association with subchondral bone) containing different phenotypes of chondrocytes and ECMs with several fiber orientations [185]. The method was based on the exploitation of a straightforward magnetic field on iron-coated streptavidin nanoparticles (10–12 nm diameter) embedded in a bioink of agarose, collagen type I and human knee articular chondrocytes (hKACs). Successfully, the parallel alignment of collagen fibers throughout the hydrogel was observed thanks to unidirectional movements derived from nanoparticles reaction to a magnet, resembling the horizontally and vertically orientation of the superficial and deep layer of the tissue to the joint; conversely, the absence of a magnetic field gave rise to the middle layer with randomly oriented collagen fibers [186]. Interestingly, chondrogenic differentiation of hKACs and GAGs content was observed in the whole assembled platform together with higher expression of collagens I and II compared to one-layer scaffold, suggesting the strong communication and coordination of cells throughout the different architectures of layers [184]. Despite the abovementioned advantages, it has to be mentioned that current commercially available 3D bioprinters still have a high cost ($10,000–150,000), low customization capacity and require costly consumables, not forgetting the necessity of the high workforce for maintenance, limiting their possible application [187].

### 4.5. Microfluidic Spinning Technology

**Figure 6:**
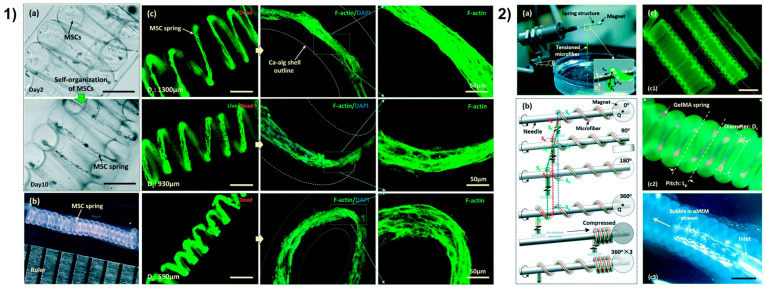
3D vSM from [196] with permission. (**1**) Self-organization of cells in GelMA spring (**a**), 13.33 length-to-diameter ratio of a MSC spring (**b**), live/dead images of MSCs spring in GelMA spring (**c**), F-actin (green) and nuclei (blue) staining of MSCs spring. Unlabeled scale bars 400 µm. (**2**) Semi-automated coiling assembly (**a**,**b**), images of helical microtubes (**c**) with various diameters (**c1**), GelMA spring (red) in the helical microtube of Ca-alginate shell (green) (**c2**), perfusion of the helical microtube (**c3**). Scale bars 800 µm.

The fourth dimension shows an additional hint for the resemble of scaffold structure for tissue engineering and modeling, but further developments are still requested to safely adapt post-printing changes with cell behavior and function [188]. Fascinating hierarchical scaffolds of blood vessels have been micropatterned by the use of templates to obtain highly interconnected and oriented microchannels that contribute to the circumferential orientation of vascular MSCs; indeed, the resemble of natural blood vessels is particularly complicated by the presence of aligned ECs in the intima and circumferentially oriented vascular smooth muscle cells (vSMCs) in the media [189]. Nevertheless, the authors reported the successful guidance by microchannels on cellular organization into multi-layered structure and elongation with circumferential orientation and contractile phenotype [190,191]. In another case, the organization of MSCs was guided by a tubular scaffold containing an outer layer of microfibers with controlled orientation and pores by electrospinning [192]. Interestingly, the still fresh microfluidic spinning technology offers more suitability, high throughput and micro-precision for the development of fiber-shaped scaffolds [126,193,194] compared to traditional approaches. This method, as a common wet spinning process, can continuously produce microfibers with a uniform diameter and spatio-temporal control due to the involvement of the science of micro-scale fluid dynamics but with an irrelevant influence by manufacturing parameters [195]; in addition, it allows to individually handle fibers and assemble fibrous structures by directly weaving and direct writing [126]. On this line, Sun et al. presented a custom 3D cell culture coaxial microfluidic system to create vascular smooth muscle (vSM)-like cellular microtubes from core-shell GelMA spring microfibers with self-organization of circumferentially oriented MSCs (Figure 6) [196]. A wet-spinning approach and semi-automated coiling method were used by Sun et al. for the production of MSCs-laden core-shell (≤160 µm diameter of inner core) GelMA microfibers and subsequently coiling and reeling into helical tubes with Calcium-alginate shell (Figure 6a,b) [196]. In this way, an appropriate pressure was circumferentially applied around the core of the springs by tailoring different parameters (i.e., the orifice size of the outlet, the flow rate of injection, the size of the helical tube etc.), resulting in the direct guidance of MSCs circumferential orientation and self-organization within the 3D environment such as vSMCs in tunica media (Figure 6, panel 1). The micro-diameter of the core allowed MSCs elongation along the longitudinal direction of the GelMA core compared to significantly less alignment at 210 µm diameter, demonstrating the importance of the microstructure size and the ability of the hydrogel to form a sufficiently small radius of curvature to induce a response on individual cells [197]. Moreover, the differentiation of MSCs in vSMCs by perfusion of differentiation media was successfully proved by the expression of related biomarkers and contractile phenotype [196]. Disparate examples concern the use of a microfluidic spinning strategy to fabricate an ultrafine core-shell nanofiber scaffold for skin regeneration [198], liver [199] and microvascular tissue engineering [200], but also for the production of free-standing porous membranes for various biomedical applications [201].

However, fibers derived by microfluidic systems are larger than electrospun fibers as the development of nanostructures has to face up the difficulties in fabricating nanoscale microfluidic chips and injecting fluids into little channels. The choice of materials is critical (only 10 materials have been used) and the fast degradation of fiber hinders its application for controlled drug delivery of small molecules. Therefore, various challenges need to be addressed to develop new biocompatible materials and extend the use of microfluidic spinning devices [126].

### 4.6. Soft Robots

Soft robots are widely investigated thanks to the combination of flexible materials with robotics and for their clear superiority in mimicking biological behaviors [202,203]. Indeed, these systems are adaptable to the simulation of specific organism-related behaviors [204] also due to the responsive guide derived by the incorporation of smart drivers (e.g., thermal signals [205], magnetism [206], light [207] and living tissue [208]). Undoubtedly, these advantages make soft robots important for several biomedical applications including target therapy [209] and drug delivery [210]. However, this fresh approach currently exploits simple materials and no-micropatterned architectures, making further advancements necessary. Sun et al. [211], inspired by the natural crawling mechanism of snakes and caterpillars, created an innovative cardiomyocytes-based soft robot for the improvement of cell contraction. The robot, composed by claws and a cardiomyocytes-laden carbon nanotube (CNT)-driven GelMA layer, was fabricated by a polyethylene substrate acting as claws template and the deposition of a magnetic nanoparticles-laden hydrogel to induce the formation of parallel-aligned CNT GelMA layer. Moreover, the status of the soft robot was monitored by an indicator-layer whose silica nanoparticles changed color in case of detection of robot deformation. Interestingly, the CNT layer showed electrical properties that induced cardiomyocytes contraction, claws drove the directional movements of the robot and the color indicator monitored cell viability. Undoubtedly, this is an example of a great potential concept for *in vitro* modeling for the most varied biomedical applications. 

## 5. Future Perspectives

The review is a roundup of the most advanced and emerging technologies for the development of *in vitro* multi-dimensional culture systems. Current available models varying from the single use to multiple integrated approaches highlight the importance of combining different 3D tools and technologies to validate bioengineered *in vitro* models able to capture the complexity of the 3D microenvironment of normal and pathological tissues. In this regard, microfluidics, multi-functional micro-/nano-material integration, bioprinting technology and innovative scaffolds fabrication have been recently introduced for high-throughput production of 3D culture models with high capability of recapitulating *in vitro* relevant biological cues of tissues. Recently, 4D cell culture technology is emerging as a powerful technology that will enable scientists to better mimic the dynamism and complexity of the human body. In fact, thanks to the possibility to tailor simultaneously multiple functionalities of the materials, such as tunable bioactivities and remote control of the physico-chemical features, 4D cell culture technology represents a disruptive progress that will arise a new era of the *in vitro* study reducing the gap between the *in vitro* and the *in vivo* [212]. Nevertheless, the research in these fields is still in its infancy and further advances in the currently available technologies are highly advisable in order to maximize their advantages and overcome the related limitations briefly summarized in Table 2. This is only possible through interdisciplinary collaborations, where the knowledge from biological sciences is combined with the engineering and designing of multifunctional, bioactive and responsive biomaterials for 3D and 4D cell cultures. Hopefully, these advanced in multi-dimensional models are expected to reduce the time need for drug approval on the market, overall cost and the need of animals for preclinical trials, rendering scientific research faster and more effective.

## Figures and Tables

**Table 1:** Details of organs-on-a-chip models reported in Section 3.2.

Organ	Cells Population	Biomaterial(s)	Purpose of the Research	References
Heart	Primary cardiomyocytes	Polycaprolactone (PCL), fibrin	Contractive cardiac tissue model	[109]
Vessels	HUVECs; human dermal fibroblasts	GelMA	Thrombosis model	[108]
Heart	HUVECs, hiPSCs	GelMA, alginate	Drug screening	[110]
Lung	Human dermal microvascular endothelial cells, human lung fibroblast	Collagen type I, PCL	Airway epithelium model	[111]
Eye	Human corneal and conjunctival cells	GelMA	Dry-eye disease model, drug screening	[112]
GI tract	Human epithelial colorectal cell line Caco-2, non-cancerous colonic cell line CCD-18Co	Polycarbonate	Gastrointestinal microbiome model	[113]
Kidney	Proximal tubule epithelial cells, renal cancer cell line A498	Gelatin, fibrin, Pluronic^®^ F127	Renal proximal tubules model	[114]
Bone	Human CD34+, nBM-MSCs, HUVECs	Fibrin	Investigation of myelothroid toxiciy	[115]
Bone	MDA-MB-231 breast cancer cells, bone marrow stem cells, endothelial cells	Decellularized bone matrix	Investigation of metastatic colonization	[117]
Bone	B cell acute lymphoblastic leukemia cell lines	PDMS	Leukemia model	[118]
Brain	Patient-derived cancer cells, HUVECs	Decellularized pig brain matrix	Glioblastoma model	[120]
Brain	Human brain microvascular endothelial cells, tumor associated macrophages, patient-derived cancer cells, human CD8+ T-cells	Hyaluronan	Glioblastoma model, chemotherapy testing	[122]

**Table 2:** A table resuming the advantages and disadvantages of the three 3D approaches explored in the review is reported.

Advantages	Disadvantages
MULTICELLULAR SPHEROIDS	
3D cell distribution, control on cell arrangement [22,45,46,47,48,53] High reproducibility [15] Cost-effectiveness [15] Few reagents [15] Easy high throughput production and scaling up [55,56,69] CSCs enrichment-method [72,73,74,78]	Absence of extracellular matrix [17] Variable size and shape [17,18,34,35] Poor control on cell functions within the spheroid [17] Inhomogeneous distribution of nutrients and gas [17] Compact cell arrangement [17]
ORGANOIDS	
Presence of basilar anatomic microstructure and cells functions [82,83,84,85,88,89] Possibility to combine cell layers of tissue-specific cell types [82,93,94,95] High cell density of systems culture [87]	Small size [86] Inadequate nutrients, factor gradients and gases supply to cells [86,87] Improper removal of cells waste products [86,87] Poor reproducibility [86] Lack of vascularization [96]
ORGAN-ON-A-CHIP	
Presence of cell–cell interactions [86,97] Presence of spatio-temporal gradients of chemicals [86,97,120,121] Proper mechanical strain [86,97] Presence of vasculature [107,108,109,110,112,113,114,115,117,118] High cell density of systems culture [87]	Poor reproducibility [97] High costs and time-consuming methodology [97]
NANOSTRUCTURED BIOMATERIALS	
Presence of 3D extracellular matrix [123,125,126] 3D cell distribution and arrangement [123,125,126,131,183,184,185,186,192,193,194] Tailoring of physico/chemical features (from nano to macro) [130,131,155,166,167,175]	Variable scaling up [127,152,153,164,165] Variable expensiveness in dependence on the technique [127,152,153,189]

## Data Availability

Not applicable.
